# Supplementary material for: Cholera in Kenya: A scoping review of current research, evidence gaps and future directions
Source: PLOS Glob Public Health. 2026 Mar 31;6(3):e0006011. doi: 10.1371/journal.pgph.0006011 (PMC13038109; doi:10.1371/journal.pgph.0006011)
Supplement: Attachment — Submitted filename: response to reviewers.docx [file pgph.0006011.s005.docx]

**Response to reviewers**

We thank the reviewers for their careful reading of the manuscript and their constructive comments, which have substantially improved the clarity, transparency, and methodological rigour of this scoping review. We have revised the manuscript accordingly, and all changes are described below with references to the relevant sections.

**Reviewer #1: The review is important to improve outcomes on cholera surveillance and response. However, there are a number of critical issues that must be addressed to ensure the manuscript conforms to the standard of scientific writing and scoping review.**

1. Certain sections were omitted, e.g., Quality assessment and Data analysis

***Quality assessment -*** *As this study is a scoping review, we did not conduct a formal critical appraisal or risk-of-bias assessment. This decision is now clearly justified and documented in* ***Section 2.4: Protocol registration and quality assessment,*** *where we explain that the primary objective was to map the breadth, scope, and evolution of cholera research in Kenya rather than to assess the validity or effectiveness of individual studies, in accordance with PRISMA-ScR recommendations.*

***Data analysis -*** *We have clarified that data were analysed using descriptive and thematic synthesis, including thematic categorisation, frequency counts, and temporal trend mapping. These procedures are now explicitly described in Section 2.3: Screening and Data Extraction, with further elaboration in Section 3.3: Temporal Trends, where thematic frequencies over time are visualised (Figure 2).*

2. The roles of the authors in the scooping exercise also omitted

*Author contributions have been clarified in the* ***methods section (Section 2.3: Screening and Data Extraction)****, detailing each author’s role in the study.*

3. The results and discussion sections are mixed up. The authors began discussing the findings in the result.

*The* ***results section (Section 3)*** *has been revised to focus strictly on* ***descriptive findings****, without discussing these points. Consequently, the discussion, policy implications, comparisons with regional and global literature, and explanations of observed patterns have been consolidated and expanded in the* ***discussion section (Section 4)****.*

**Reviewer #2: Given the ongoing cholera pandemic and its recurrent outbreaks in sub-Saharan Africa, it is commendable that the authors undertook a comprehensive mapping of cholera research in Kenya.**

1. For the search strategy, the query “cholera AND Kenya” across all databases is overly restrictive and likely excluded studies using alternative terminology such as “Vibrio cholerae”, “waterborne disease”, or “WASH-related cholera”. I would recommend providing the full keywords, filters and timelines used for each database, to help in reproducibility, as stated in the PRISMA-ScR Checklist (Item 8).

*The primary objective of this review was to capture the breadth of peer-reviewed research explicitly focused on cholera in Kenya. We therefore used the broad and disease-specific search term* ***“cholera AND Kenya”*** *to maximise sensitivity while maintaining specificity and consistency across databases with different indexing systems. We acknowledge that this strategy may not have captured a small number of laboratory-based or environmental studies referring exclusively to* Vibrio cholerae *without explicit mention of “cholera”.*

2. Please provide the last search date or timeframe.

*We have now explicitly stated the* ***final search date*** *in the Methods section to improve transparency and reproducibility, in line with PRISMA-ScR recommendations. Specifically, we have added the following information to* ***Section 2.1: Search strategy****:*

*“The final database searches were conducted on* ***8 October 2024****.”*

3. The authors mentioned the systematic search of five databases, including Google Scholar, Web of Science, PubMed, Embase, and Scopus. However, in the PRISMA flow diagram (Figure 1), there is no data for Google Scholar.

*Google Scholar returned* ***more than 50,000 records*** *for the search term* “cholera AND Kenya”*, making comprehensive export and de-duplication infeasible.*

4. The use of Rayyan is recognized. However, reviewer roles, conflict resolution, and data extraction validation are not stated.

*We have revised* ***Section 2.3: Screening and Data Extraction*** *to include author roles in the study.*

5. The authors mentioned the inclusion of non-primary studies, such as reviews, but stated “ineligible study design” as a reason for exclusion in Figure 1. A clarification on this is could be beneficial.

*Records classified as having an “ineligible study design” referred to non-research publication types, including editorials, commentaries, opinion pieces, protocols, news items, and conference abstracts without full empirical data. We have explicitly stated this in figure 1 legend.*

6. For each included study, the authors should present the characteristics of the data charted with respective citations in a table.

*The characteristics of each included study were systematically charted and are presented in* ***Supplementary Table 1,*** *which serves as the data charting table for this scoping review.*

*Supplementary Table 1 provides, for each included study, the study citation (authors, year, and journal), study objectives, design and methods, geographic setting, thematic classification, key findings, identified research gaps, challenges, and limitations. This table was generated using a standardised data extraction form, as described in* ***Section 2.3: Screening and Data Extraction.***

*To improve clarity, we have now explicitly referred to Supplementary Table 1 as the* ***data charting table*** *in the Methods section and clarified its role in documenting study characteristics.*

7. In section 3.2, the authors provide an informative table which shows the geographic focus of the studies across multiple countries, including Kenya. For a scoping review centered on Kenya, a similar table or map that shows the distribution of studies/ data on the county-level could be added.

*We agree that a county-level visualisation could provide additional geographic granularity. However, we chose not to include a county-level table or map for the following reasons.*

*First, a substantial proportion of the included studies were* ***multi-site, national-level, or regional analyses****, or did not report data at consistent county resolution. In many cases, geographic identifiers varied across studies (e.g., counties, provinces, districts, refugee settlements, or water bodies), limiting comparability and increasing the risk of misclassification.*

*Second, the primary objective of this scoping review was to* ***map the thematic scope, methodological approaches, and temporal evolution*** *of cholera research in Kenya, rather than to provide a fine-scale spatial synthesis of study locations. Presenting county-level distributions based on heterogeneous and incomplete geographic reporting could therefore introduce unintended bias or imply a level of spatial precision not supported by the underlying data.*

*We have added a brief clarification in the* ***Discussion (Limitations section)*** *acknowledging that finer-scale spatial synthesis was not undertaken and represents an important area for future work.*

8. Themes such as mortality and risk factors of cholera could be explored and discussed further to strengthen the manuscript.

*We opted not to introduce mortality and risk factors as separate thematic categories, as these concepts cut across multiple domains and are more appropriately synthesised within epidemiological, environmental, and social frameworks. This approach preserves thematic coherence while strengthening interpretive depth.*

*To strengthen clarity and emphasis, we have* ***expanded the discussion of mortality and risk factors*** *within existing thematic sections rather than introducing new standalone themes. Specifically:*

- ***Mortality patterns and determinants*** *are discussed in* ***Section 3.4.1 (Epidemiology, Surveillance, and Disease Modelling)*** *and synthesised further in the* ***Discussion (Section 4),*** *including links to delayed care-seeking, surveillance limitations, and health-system constraints.*
- ***Risk factors for cholera transmission****, including WASH deficiencies, environmental contamination, climate variability, behavioural practices, and socioeconomic vulnerability, are elaborated across* ***Sections 3.4.1 (Epidemiology), 3.4.2 (Environmental Determinants and WASH),*** *and* ***3.4.7 (Social, Behavioural, and Economic Aspects)****, and integrated in the discussion in Section 4.*

9. The Results-Discussion boundary seems blurred. Discussion begins to appear within “Future directions” paragraphs under each theme. I would recommend that the authors consolidate all “Future directions” into a single Discussion summarising what is known and unknown.

*We have revised the manuscript to* ***clarify the “Results-Discussion” boundary*** *while preserving the thematic structure of the scoping review.*
